# Smokers’ Engagement Behavior on Facebook: Verbalizing and Visual Expressing the Smoking Cessation Process

**DOI:** 10.3390/ijerph19169983

**Published:** 2022-08-12

**Authors:** Jezdancher Watti, Máté Millner, Kata Siklósi, Hedvig Kiss, Oguz Kelemen, Dávid Pócs

**Affiliations:** Department of Behavioral Sciences, Albert Szent-Györgyi Medical School, University of Szeged, 6722 Szeged, Hungary

**Keywords:** Facebook, engagement, smoker, smoking, smoking cessation, public health, Facebook reaction, comment, motivational interviewing, processes of change

## Abstract

The “processes of change” and “motivational language” are common in smoker Facebook users’ comments under smoking cessation support contents. Smokers can combine this verbalization of the smoking cessation process with visual expression when they use comments and Facebook reactions at the same time. The aim of this study was to understand the relationship between processes of change, motivational language, and the Facebook reaction buttons. A total of 821 smokers’ comments were analyzed in the current study (*n* = 821), which responded to image-based smoking cessation support contents. The processes of change and the motivational language used in the investigated comments were identified. These linguistic categories were compared with the usage of reaction buttons. The Facebook users who used the “Haha” reaction button wrote a significantly higher proportion of sustain talk than those who used the “Like” or “Love” reaction buttons. The Facebook users who combined the comment and “Love” reaction wrote significantly more change talk than those who did not utilize these buttons. We suggest that the “Haha” reaction may be a negative indicator, the “Like” reaction may be a neutral indicator, and the “Love” reaction may be a positive engagement indicator in terms of the smoking cessation process during Facebook-based interventions. These results may highlight how to evaluate Facebook reactions relating to smoking cessation support contents.

## 1. Introduction

### 1.1. Engagement Indicators on Facebook

Worldwide, the creative integration of technology to address health problems is gaining importance. Digital interventions can be used to drive critical behavior change processes that leads to improved health behaviors [1]. “Engagement” with digital behavior change interventions plays an important role in their effectiveness [1,2,3,4,5]. In behavioral science literature, “engagement as usage” phrase suggests that engagement can involve the quantity of usage (e.g., frequency, duration) and the quality of usage (e.g., use of specific buttons) [1,2,6,7,8,9]. This viewpoint can be advantageous in health behavior change during public health campaigns [9,10,11,12]. Both the quantity and the quality of usage can be associated with health behavior change, and they can differ in internet-based interventions using different platforms [11,12].

Facebook is a popular and extensively available platform, which could be highly applicable for public health interventions among adolescents and adults [13,14,15]. The quality of usage on Facebook includes the users’ interactions, which are actions on certain buttons that the person uses in relation to a given Facebook post [16,17,18,19,20]. For example, the “comment” interaction button is used for writing a text or publishing an image under the social media content [21,22]. A high number of comments are associated with health behavior change [23,24]. However, the content analysis of comments on public health Facebook posts is an area barely researched.

Further popular interaction buttons are Facebook “reactions” [18,24]. They can give users the possibility to express their emotions and can have a particular function in public health interventions [16,19,20]. According to a tobacco cessation study, getting one “Like” reaction on an intervention platform can be linked with smoking reduction by roughly one cigarette per week [25]. Until now, there have been only a few quantitative analyses of “Love”, “Haha”, “Wow”, “Sad”, or “Angry” reactions in public health interventions [19,20]. Facebook-based smoking cessation interventions which involve using reaction buttons or writing comments indicate engagement [24,25]. This can explain why it is important to understand how reaction buttons are associated with commenting.

In the current study, “comment” and “reaction” buttons were used as engagement indicators. The combination of these interactions during Facebook-based smoking cessation intervention is still undetermined. In particular, it is not known which Facebook reaction buttons may be related to the smoking cessation process expressed in the users’ comments. Two psychological approaches are used widely to examine the verbalization of smoking cessation: “processes of change” (as a part of the ‘Transtheoretical Model’) and “motivational language” (as a part of ‘Motivational Interviewing’) [26,27,28].

### 1.2. Processes of Change

The Transtheoretical Model is an integrative theory of various psychotherapies, which provides processes of change to understand how shifts in health behavior occur [29]. The processes of change are specific actions used by people to change their health behavior [30]. There are two groups of processes. Experiential processes are cognitive, affective, and evaluative actions, which are taken primarily in the early stage of behavioral change (e.g., “consciousness raising”, “dramatic relief”, or “self-reevaluation”) [30,31,32]. In contrast, behavioral processes are action-oriented, and used primarily in a later stage of behavioral change (e.g., “reinforcement management” or “stimulus control”) [30,31,32].

The Transtheoretical Model is frequently used to design internet-based public health interventions and was found to result in significant effects on health behavior changes, including smoking cessation [33,34]. However, only a few studies performed a quantitative analysis of the association between the processes of change and the engagement on Facebook. A previous study of smoking cessation support has reported that Facebook posts utilizing “dramatic relief” and “self-liberation” generated comments below the average number, while Facebook posts based on “consciousness raising” elicited comments above the average number among smokers [35]. However, no studies have described the way Facebook users express the processes of change in combination with the reaction buttons, Therefore, one aim of this study was to explore this relationship. Writing comments without the usage of reaction buttons was the comparison group.

The research question related to the Transtheoretical Model is the following:

- How do smokers express the processes of change in combination with Facebook reaction buttons?

### 1.3. Motivational Language

Motivational Interviewing is a directive, client-centered counseling approach, which hypothesizes that certain verbal utterances (“motivational language”) can be predictors of health behavior change [36,37,38,39,40]. These motivational utterances include two major linguistic categories: “change talk” and “sustain talk” [36,37]. The “change talk” linguistic category includes phrases that move someone toward a preferable behavioral change, which is linked to improvements in behavioral outcomes [38,39]. If the client uses more change talk, this can predict successful behavioral change [39]. On the other hand, the “sustain talk” category contains phrases that discourage movement toward a change goal, but favor maintaining the status quo, which is a detrimental behavior in our scenario [36,40,41].

Motivational Interviewing is an evidence-based method of treating and preventing addictions during face-to-face interventions, as well as other alternative approaches [42]. However, findings on internet-based Motivational Interviewing are controversial, with informal promising results in treating alcoholism but inconsistent results in smoking cessation [42]. There is insufficient evidence to show whether Motivational Interviewing can help people to stop smoking [43] or increase adherence to smoking cessation medications [44]. So far, there has been little discussion about the effect of Facebook-based Motivational Interviewing on smoking cessation outcomes. In the past a study sought to explore which Motivational Interviewing strategies increase engagement, and which strategies decrease it [45]. “Relational Motivational Interviewing” strategies may achieve higher engagement rate, “Elaborating Change Talk” strategies can elicit more change talk, while “Affirming Change Talk” strategies tend to obtain higher fan-total reach ratio [45]. However, no previous study has investigated the relationship between the motivational language and Facebook reaction buttons, which was one of our study goals. The comparison group included writing comments without the usage of reaction buttons.

The research question related to Motivational Interviewing is:

- How do smokers express the motivational language in combination with Facebook reaction buttons?

## 2. Materials and Methods

### 2.1. Participants

The participants of the present study were Facebook users who wrote comments to social media contents of the Hungarian “Cigarette break” Facebook page. “Cigarette break” is a non-profit, internet-based smoking cessation intervention operated by the authors and other experts, students, and lecturers at the University of Szeged. This Facebook page avoids intimidating and judgmental contents and seeks to support cessation based on the motivational interviewing counseling approach [46]. The primary aim of this Facebook-based intervention was to directly support smoking cessation. The secondary aim was relapse prevention aimed at former smokers. Finally, the third aim was to improve non-smokers’ social support skills. This can indirectly improve smoking cessation outcomes through partner support. Finally, our researcher identity is transparent on the Facebook page. We will keep the users of the Facebook page informed about the current research and its results.

The demographic data of reached users are made available to the page administrators by Facebook. We exported the cumulative and anonymized demographic data from the “Facebook Insights” database on 14 September 2020. These data are subject to the privacy policies of Facebook, and they are provided to the page administrators by Facebook with the consent of the users. On this day, 10,227 people liked the investigated Facebook page. Of them, 53% were women and 47% men, 2% were between 13 and 17 years old, 84% were between 18 and 35 years old, and 14% were older than 35 years. Furthermore, 96% of them indicated Hungary as their location on their Facebook profile.

### 2.2. Stimuli

The focus of the study was the analysis of the Facebook users’ interactions. Hence in this chapter the common stimulus to which participants responded with comments and reaction buttons is described. In all, 1294 social media contents were published between 7 March 2017 and 14 September 2020. First, we excluded 150 contents which were not related to cessation (e.g., administrators’ Facebook posts), as we wanted to explore the Facebook users’ response to contents which support smoking cessation. Then, we excluded 45 contents which were not adherent to motivational interviewing or were not image-based. This was necessary since the investigated Facebook-based intervention relied on contents which were adherent to the motivational interviewing approach and contained an image, as described in our previous research [45]. The next step of the exclusion process was to select Facebook posts which received comments related to smoking cessation. Of the remaining 1099 Facebook posts, 621 (57%) received no comments, and 300 (27%) had comments which were not related to cessation.

In total, 178 Facebook posts were included in the current study. These posts received comment which were related to cessation and those which were not related to cessation. It is also important to highlight that only the original Facebook posts published on the investigated Facebook page were included in the current study. This was necessary in order to evaluate the Facebook users’ responses to the same original stimulus. The social media contents shared by the Facebook users were ignored since, in this case, the Facebook profile of the given users may have changed the responses of other Facebook users as a new stimulus. For instance, a profile of a known person can be a positive or negative stimulus depending on the user’s attitude towards the known person (co-worker, boss, family member, famous person etc.). Overall, the stimuli applied for investigation were original Facebook posts which were adherent to motivational interviewing, contained an image, and supported smoking cessation. This content analysis can only be interpreted in this context. We show some examples of the stimuli in  File S1 in Supplementary Materials.

### 2.3. Design

The research method of the current study was hypothesis-generating, retrospective content analysis. We analyzed the Facebook users’ interactions (comments and reactions) to the same stimuli (investigated Facebook posts). The participants and the stimuli were presented earlier. The interaction data were analyzed on the comment level. A total of 821 Facebook users commented on the 178 Facebook posts included. All of the comments came from different Facebook users. Therefore, the number of items was the number of Facebook users who wrote a comment (*n* = 821). The 821 Facebook users included in the study may have been out of the 10,227 Facebook fans, or they may not have previously liked the Facebook page. The 821 Facebook users are different, so they are only included once in the database. In this subsection, the aspects of the comment analysis are presented.

Since we wanted to investigate the Facebook users’ direct response to a given content, we analyzed only the “first comments”. For example, if a Facebook user wrote “I want to quit” under the original social media content, then this was considered a first (initial) comment. If another Facebook user added “I want to quit too”, that was a second comment. Second and third comments were not included in the current analysis, as they may have been responses to first comments (as a new stimulus), and not responses to the original Facebook post (as the investigated stimulus). All of the 821 comments were first comments. We also analyzed the usage of Facebook reactions, which were also direct responses to a given content. We analyzed “first reactions” to the Facebook posts, not “second or third reactions” to comments. In this way, the relationship between “first comments” and “first reactions” was assessed at the same level (post-level). Finally, Facebook users wrote comments and used reaction buttons on this public Facebook page freely and voluntarily, without any external pressure.

We collected whether the Facebook user who wrote the comment used any Facebook reaction button related to the social media content. If the Facebook user utilized a reaction for the given cessation support content, we also recorded the type of reaction button used: ‘Like’, ‘Love’, ‘Haha’, ‘Wow’, ‘Sad’, and ‘Angry’.

The comments were classified into different categories according to the Transtheoretical Model and the Motivational Interviewing approach. The analyzed comments did not contain personal or sensitive data, and the aim of the comment analysis was to investigate the verbalization of the smoking cessation process. The processes of change are specific actions which are taken by smokers to stop tobacco use. For example, the sentence “I read about people who have successfully stopped smoking” is an experiential process (“Consciousness Raising”), while the sentence “I reward myself for small quitting steps.” is a behavioral process (“Reinforcement management”). Moreover, change talk used by smokers can express motivation for tobacco use cessation or action towards tobacco use cessation. The sentence “I wish I could quit smoking” is preparatory change talk (“desire”), while the sentence “I bought a nicotine patch” is mobilizing change talk (“taking steps”). On the other hand, sustain talk used by smokers can express demotivation for tobacco use cessation or inaction towards tobacco use cessation. Examples of sustain talk are “I like to smoke cigarettes” (“desire”), and “I smoked my friend’s cigarette” (“taking steps”). The subcategories are described in File S2 in Supplementary Materials including further examples. The original wording of the example sentences has been modified to avoid subsequent identification. In conclusion, these definitions highlight that only smokers’ phrases were classified. Thus, the strict use of definitions implies that Facebook users who write these phrases can be considered smokers based on the text. The process of comment analysis is described in the next subsection. The definitions of the comment categories are described in the Introduction section and summarized below.

Definitions of “processes of change”:

- These phrases are specific actions (experiential and behavioral processes) which are taken by smokers to stop tobacco use.
- Experiential Processes: Smokers’ phrases that express cognitive, affective, and evaluative processes are used primarily in the early stage of tobacco use cessation. These five processes are “Consciousness Raising”, “Dramatic Relief”, “Social Liberation”, “Environmental Reevaluation”, “Self-reevaluation”.
- Behavioral Processes: Smokers’ phrases that express action-oriented processes used primarily in a later stage of tobacco use cessation. These five processes are “Self-liberation”, “Reinforcement management”, “Helping Relationship”, “Stimulus Control”, “Counter Conditioning”.

Definitions of “motivational language”:

- These phrases used by smokers who express motivation/demotivation for tobacco use cessation or action/inaction towards tobacco use cessation (change talk/sustain talk).
- Change talk: Smoker’s phrases that favor movement toward tobacco use cessation. Subtypes of change talk: “desire, ability, reason, need” (DARN, “preparatory change talk”) and “commitment, activation, taking steps” (CAT, “mobilizing change talk”).
- Sustain talk: Smoker’s phrases that favor remaining a tobacco user rather than moving toward tobacco use cessation.

### 2.4. Procedure

This subsection describes the comment analysis process. Firstly, the comments were divided into two almost identical groups based on the topic. On the one hand, 53% of the comments (*n* = 439) did not contain phrases referring to smoking cessation, and this was considered a control group for comparison. For example, we included comments such as “I disagree with this post.” or “I really like this post.”. On the other hand, 47% of the comments (*n* = 382) dealt with smoking cessation, and this group was further classified. Only smokers’ comments were assessed as a cessation topic. The comments which were related to cessation and were written by non-smokers was classified as a non-cessation topic. For instance, “My husband threw away his cigarettes”—it is not clear whether the speaker is a smoker or not. This was categorized as a non-cessation topic. In the cessation-topic phrase “I threw out my cigarette” it is obvious that the person is a smoker. In summary, while non-smokers do not write comments on the psychological process of their smoking cessation, smokers can do it. Smoking status may be identified from the comment analysis.

Ten processes of change were distinguished. A comment could contain several processes of change subcategories, but only one from each subcategory. A total of 260 processes of change were identified in the analysis, of which 150 (58%) were experiential processes, and 110 (42%) behavioral processes. Hence, not all comments on cessation included processes of change. Two raters classified all of the 821 first comments separately into experiential processes, behavioral processes, and comments which were not related to cessation (Cohen kappa value of 0.935).

Comments on cessation (*n* = 382) were also categorized into change talk and sustain talk according to Motivational Interviewing. A comment could contain change talk and sustain talk separately or both. A total of 778 motivational utterances were identified in the analysis, of which 475 (61%) were change talk and 303 (39%) sustain talk. Therefore, all comments on smoking cessation could be categorized using Motivational Interviewing. Two raters classified the comments investigated separately into change talk, sustain talk, and comments which were not related to cessation (Cohen kappa value of 0.944). Comments on cessation may contain more than one process of change, change talk, or sustain talk. It may have been the case that the same text fell into both linguistic categories of process of change and change talk, but this combination happened rarely. This fact highlights the similarities between the two psychological theories. However, the low number of texts in both linguistic categories did not affect the statistical analysis.

The study groups were defined as the combination of writing comments and using certain reaction buttons. The comparison group included writing comments without the usage of reaction buttons. After the classification of comments, statistical analyses were carried out. Pearson’s chi-square test was used to compare these categorical variables, and the effect size was measured by Cramer’s V value. As the number of processes of change and motivational utterances were not normally distributed, non-parametric statistical tests were used. The Kruskal-Wallis H test with Dunn’s test was utilized to test for reaction buttons. In both cases, the effect size was measured by eta squared for comparison. All analyses were conducted using the Statistical Package for the Social Sciences software. The *p* value of less than 0.05 was taken to indicate a significant effect, and the *p* value of less than 0.001 was taken to indicate a highly significant effect. The original data are available in File S3 in Supplementary Materials.

## 3. Results

### 3.1. Trends in Comments and Reactions

In this subsection, we present the number and percentage distribution of comments in the different categories, summarized in Table 1. About 20% of the Facebook users who wrote a comment used one of the reaction buttons, while the majority (80%) did not combine commenting with Facebook reactions. There was no significant difference between the cessation-related and non-cessation-related comments regarding the combination of comments with the use of the reaction buttons (20.4%, 20.7%). Due to the low proportion of combinations, statistical analysis of the six different reaction types was not feasible, however, some trends can be observed. For example, cessation-related comments were combined with remarkably fewer “Haha” reactions (3.9%) than comments which were not related to cessation (6.2%). There was no significant difference in the combinations based on the Transtheoretical Model and Motivational Interviewing. However, tendencies can also be observed. Compared to the combination rate of the control group (20.7%), it was lower for comments having experiential processes and sustain talk (14.7%, 15.8%) and higher for statements containing behavioral processes and change talk. (26.9%, 22.5%). In view of all that has been mentioned so far, there was no significant difference between the investigated comment categories in terms of the combination of comments with the use of the reaction buttons. However, this classification did not consider the number of processes of change or motivational utterances identified in a comment. Therefore, in the following chapters, we compare the number of processes of change and motivational utterances by combinations.

### 3.2. Comment Analysis Based on the Transtheoretical Model

In this subsection, we compare the number of processes of change by reaction button usage among cessation-related comments, which is summarized in Table 2. We analyzed only classified comments on smoking cessation from which it could be clearly established that the commenter was a smoker.

Our research question about the Transtheoretical Model was “How do smokers express the processes of change in combination with Facebook reaction buttons?”. Our results show that Facebook users who utilized a certain type of reaction button did not use significantly more experiential processes or behavioral processes than those who did not use Facebook reactions in combination with their comment. In this analysis, we found only one significant difference in the total number of processes applying the Kruskal-Wallis H test (χ^2^(3) = 8.347, *p* = 0.039, η^2^ = 0.014). Dunn’s pairwise test was performed to identify the significant difference. Thus, we found that those who combined their comment with “Love” reaction used significantly more processes of change than those who combined their comment with “Haha” reaction, 1.10 (SD: 0.74) and 0.33 (SD: 0.49), respectively. Although the Dunn’s pairwise test did not identify a significant difference between those who used a reaction combination and those who did not. It is worth highlighting a trend which is nearly significant (*p* = 0.066). Compared to all processes of change, smokers utilizing the reaction buttons used fewer experiential processes (61% vs. 52-31-20%) and more behavioral processes (39% vs. 48-69-80%) than those who did not used Facebook reactions in combination with their comment. In summary, we found only one notable association between the number of processes of change and the usage of Facebook reactions.

The above results provide a partial answer to the question of which psychological model can better explore the relationship between the verbalization of health behavior change and the usage of Facebook reactions: The Transtheoretical Model or the Motivational Interviewing Approach. The Transtheoretical Model alone cannot be entirely suitable for exploring the association between health behavior change and the usage of Facebook reactions.

### 3.3. Comment Analysis Based on Motivational Interviewing

In this subsection, we compare the number of motivational utterances by reaction button use among cessation-related comments, which are summarized in Table 3. Our research question was “How do smokers express the motivational language in combination with Facebook reaction buttons?”. Our results suggest that Facebook users who wrote a large number of motivational utterances and change talk combined their comments with “Love” reactions. We found a significant difference in the number of motivational utterances among those who used reaction buttons applying Kruskal-Wallis H test (χ^2^(3) = 12.825, *p* = 0.005, η^2^ = 0.026). The number of motivational utterances was the highest among those who utilized the combination of comment and “Love” reaction (mean: 3.10, SD: 1.37). Dunn’s pairwise test revealed that this value was significantly higher compared to the “Haha” reaction combination (mean: 1.47, SD: 0.74, *p* = 0.006), the “Like” reaction combination (mean: 1.69, SD: 1.08, *p* = 0.006), and among those not using the reaction combination (mean: 1.78, SD: 1.03, *p* = 0.007). We found a similar significant difference in the amount of change talk (χ^2^(3) = 19.243, *p* < 0.001, η^2^ = 0.043). The amount of change talk was also outstanding among those who utilized the combination of comment and “Love” reaction (mean: 2.60, SD: 0.97). Dunn’s pairwise test confirmed that this value was significantly higher compared to the “Haha” (mean: 0.67, SD: 0.72, *p* < 0.001) and “Like” reaction combination (mean: 1.24, SD: 0.78, *p* = 0.006), and among those who did not use them (mean: 1.24, SD: 0.99, *p* = 0.001). In summary, the combination of comment and “Love” reaction is characterized by a high number of motivational utterances and change talk.

The proportion of motivational language subcategories was also compared to the usage of reaction buttons. We found some significant differences (χ^2^(3) = 11.116, *p* = 0.011, η^2^ = 0.022). This also answers our research question, “How do smokers express the motivational language in combination with Facebook reaction buttons?”. Compared to all motivational utterances, smokers using the “Haha” reaction button wrote a significantly lower proportion of change talk (40%) than those having the “Like” (76%, *p* = 0.026) or “Love” reactions (89%, *p* = 0.042). On the other hand, this means that those who used the “Haha” reaction button wrote a significantly higher proportion of sustain talk (60%) than those who used the “Like” (24%, *p* = 0.026) or “Love” reaction (11%, *p* = 0.042). Overall, the combination of comment and “Haha” reaction is characterized by a high proportion of sustain talk.

Comparing Table 2 with Table 3, we found more significant associations between motivational language and reactions than processes of change and reactions. Therefore, motivational language seems to be more suitable for exploring the relationship between the verbalization of the smoking cessation process and the usage of Facebook reactions.

## 4. Discussion

### 4.1. Principal Results

The primary aim in this research was to understand the relationship between the usage of Facebook reaction buttons and the verbalization of the smoking cessation process (“processes of change”, “motivational language”) during a Facebook-based intervention. We did not find a significant relationship between comments and Facebook reaction combinations in terms of whether the comment included processes of change or motivational utterances or if it was related to cessation or not. However, we identified several significant associations between the number of motivational utterances and the usage of Facebook reactions. Those who combined the comment and “Love” reaction wrote significantly more motivational utterances and change talk than those who used the “Haha” and “Like” reactions or those who did not utilize these buttons at all. In addition, those who combined the comment and “Haha” reactions were characterized by a high proportion of sustain talk. Smokers can express change talk with the “Love” reaction, and sustain talk with the “Haha” reaction, when reacting to Facebook posts supporting smoking cessation. This seems to be an important result in this area since Facebook-based smoking cessation interventions usually use reaction buttons as a common indicator of engagement, regardless of their emotional background or meaning [24,25,47].

This assumption can also be confirmed by a significant correlation of these reactions with the number of processes of change. In this case, the ambivalence of the smoking cessation process may be manifested in a remarkably high and markedly low number of processes of change. The “Love” reaction was associated with an outstanding number of processes of change, while among those using the “Haha” reaction, the processes of change were conspicuously low. This difference was found to be significant. However, in terms of the number of processes of change, we did not find a significant difference between those who used the reaction button and those who did not utilize it. Therefore, based on our results, motivational language is more effective in exploring the relationship between verbalization of the smoking cessation process and usage of Facebook reactions than any other processes of change.

### 4.2. Hypotheses for Future Research

This was an exploratory, hypothesis-generating study designed to obtain insights into the relationship between Facebook reaction buttons, processes of change, and motivational language. Firstly, the hypotheses related to Facebook reaction buttons are discussed. Our results suggest that the “Love” reaction can be associated with a high number of change talk and a large proportion of change talk during Facebook-based smoking cessation interventions. Hence, using the “Love” response can be a visual expression of the smoking cessation process. This suggests that the relationship between the “Love” reaction and behavioral outcomes (e.g., the duration of smoking cessation) could be an exciting area of research. Presumably, as with change talk, the “Love” reaction could be a positive predictor of smoking cessation, and possibly other health behavior changes. Another visual expression of the smoking cessation process could be the use of the “Haha” response. Our results highlight that the “Haha” reaction is more likely to be associated with a large proportion of sustain talk during Facebook-based smoking cessation interventions. Therefore, for future research, we propose a longitudinal study of the “Haha” reaction as a negative predictor of the smoking cessation process.

Finally, our research also revealed that the “Like” reaction may not have a specific role in the expression of the smoking cessation process during Facebook-based interventions. Those who used the “Like” reaction button, and those who did not used reaction buttons, wrote almost the same number and proportion of linguistic categories. This contrasts with the theoretical assumption that utilizing a combination of comment and “Like” reactions may imply a higher level of engagement than writing only a comment [48,49]. Some studies classified the “Like” reaction as a positive engagement indicator since it seems to express positive feelings [20,49]. Other studies revealed that the quality of the “Like” reaction as an engagement indicator can be highly context-dependent [19,50]. Our research shows that in practice the “Like” response may be not relevant for the smoking cessation process. This is an important finding since the “Like” response is considered as an indicator of engagement of smoking cessation interventions. Further investigation of this issue is needed in the future. In addition, the low number of “Wow”, “Sad” and “Angry” reactions did not allow for their detailed analysis in this study, but we propose to investigate these in relation to their role in the visual expression of the smoking cessation process using a methodology similar to the one applied in our study. On the other hand, the usage of reaction buttons may be context dependent. For example, the “Sad” reaction in addictology may indicate a resistance to the smoking cessation intervention, whereas in cancer prevention it may express Facebook users’ engagement as a response to stories by cancer patients [19].

Hypotheses for future testing regarding to reaction buttons:

- The “Love” reaction can be associated with a high number of change talk and a large proportion of change talk during Facebook-based smoking cessation interventions.
- The “Haha” reaction can be associated with a large proportion of sustain talk during Facebook-based smoking cessation interventions.
- The “Like” reaction may not have a specific role in the expression of the smoking cessation process during Facebook-based interventions.

### 4.3. Limitations and Strengths

Some limitations of the study should be mentioned. Firstly, the sociodemographic data and smoking habits of the participants are not known precisely. This is why sociodemographic data and smoking habits may be sources of bias. Therefore, the findings may not be representative in other settings. Secondly, due to the low number of elements, we were not able to analyze the “Wow”, “Sad” and “Angry” reaction buttons. The scarce use of these reaction buttons may be attributed to the fact that the stimuli under study were Facebook posts which supported smoking cessation relying on motivational interviewing. At its core, Motivational Interviewing is a non-confrontational approach which respects smokers’ autonomy and can lead to a low number of such negative emotional responses. It is therefore important to emphasize that our results are primarily evaluated in relation to the stimulus under investigation. The investigated Facebook posts were based on the Motivational Interviewing approach, which may have influenced the responses of Facebook users (using “motivational language” rather than “processes of change”). A previous study showed that the usage of Motivational Interviewing can elicit significantly more “change talk” in smokers [51]. Therefore, future studies should also analyze Facebook posts which do not be based on the Motivational Interviewing approach. The third limitation may be that a regression-based model was not conducted. However, regression analysis cannot be used since there are not enough variables to apply it. The averages of some psychological terms were compared in the study groups. These groups were defined as a combination of writing comments and using certain reaction buttons. It follows from the grouping that the findings in the current study cannot be used to make causal inferences. It is not known whether Facebook users wrote comments first and then used reaction buttons or vice versa. Lastly, another limitation is social-desirability bias. The engagement on Facebook is potentially confounded by socially desirable responding. For example, the infrequent use of “Wow”, “Sad”, and “Angry” reactions can relate to social desirability bias, particularly if the users know each other or can easily identify each other outside of Facebook.

A major strength of the study is the length of the study period (three and a half years), which allowed us to select a large sample of Facebook posts (1294 posts). The long study period was also necessary since 16% of the posts received a comment about cessation. Smokers may find it difficult to describe their thoughts and experiences of quitting on a public Facebook page. Therefore, the selected database of 178 Facebook posts is considered valuable. Another strength of the research is that instead of using a questionnaire method, we examined users’ responses to a Facebook-based cessation support intervention in real-life conditions. Furthermore, the correlations between the comments and the reaction buttons were analyzed at the level of the comments rather than at the level of the posts. Thus, the combinations of the comments with the reaction buttons were reliably identified in a real intervention context.

## 5. Conclusions

Different conclusions can be drawn from the research findings to help health professionals assess the engagement indicators of the smoking cessation process during Facebook-based interventions. This is the first study to suggest that the “Like” response may not be a specific engagement indicator for the smoking cessation process. In contrast, the “Love” and “Haha” reactions may be specific engagement indicators and may express the ambivalence of the smoking cessation process visually. At the Facebook post level, smokers who want to quit can use “Love” reaction, while smokers who do not want to quit can use “Haha” reaction. These results are interpretable in the investigated online context (smoking cessation support contents as stimuli). This is supported by the fact that the number of change talk, the proportion of change talk, and the number of processes of change were significantly higher among those using the “Love” response, while the proportion of sustain talk was higher and the number of processes of change were significantly lower among those having the “Haha” response. During motivational interviewing, the consultant uses different communication strategies based on the client’s verbal responses (change talk and sustain talk). Our results suggest that “Love” and “Haha” reactions, as the client’s visual responses, may also be suitable for selecting these strategies in this online context. In summary, in terms of the health behavior change, the “Haha” reaction may be a negative engagement indicator, the “Like” reaction may be a neutral engagement indicator, and the “Love” reaction may be a positive engagement indicator. Therefore, the findings of this study suggest that measuring and analyzing the processes of change and motivational utterances in Facebook users’ comments can be valuable for Facebook-based smoking cessation interventions.

## Figures and Tables

**Table 1:** The number (*n*) and the distribution (%) of comments in different categories according to the Transtheoretical Model and Motivational Interviewing (*n* = 821).

	Comments, *n* (%)							
	Combination of Comment and Reaction		Types of the Combined Facebook Reaction					
	Non-Combined	Combined	Like	Love	Haha	Wow	Sad	Angry
Comments by topic								
Cessation Topic								
(*n* = 382)	304 (79.6)	78 (20.4)	49 (12.8)	10 (2.6)	15 (3.9)	1 (0.3)	0 (0.0)	3 (0.8)
Non-cessation Topic (Control Group)								
(*n* = 439)	348 (79.3)	91 (20.7)	47 (10.7)	9 (2.1)	27 (6.2)	4 (0.9)	2 (0.5)	2 (0.5)
Comments by “Transtheoretical Model”								
Experiential Processes								
(*n* = 109)	93 (85.3)	16 (14.7)	13 (11.9)	1 (0.9)	1 (0.9)	0 (0.0)	0 (0.0)	1 (0.9)
Behavioral Processes								
(*n* = 78)	57 (73.1)	21 (26.9)	12 (15.4)	4 (5.1)	4 (5.1)	0 (0.0)	0 (0.0)	1 (1.3)
Experiential Processes and Behavioral Processes								
(*n* = 23)	17 (73.9)	6 (26.1)	3 (13.0)	3 (13.0)	0 (0.0)	0 (0.0)	0 (0.0)	0 (0.0)
Non-cessation Topic (Control Group)								
(*n* = 439)	348 (79.3)	91 (20.7)	47 (10.7)	9 (2.1)	27 (6.2)	4 (0.9)	2 (0.5)	2 (0.5)
Comments by “Motivational Interviewing”								
Change Talk								
(*n* = 204)	158 (77.5)	46 (22.5)	32 (15.7)	7 (3.4)	5 (2.5)	1 (0.5)	0 (0.0)	1 (0.5)
Sustain Talk								
(*n* = 95)	80 (84.2)	15 (15.8)	7 (7.4)	0 (0.0)	6 (6.3)	0 (0.0)	0 (0.0)	2 (2.1)
Change Talk and Sustain Talk								
(*n* = 83)	66 (79.5)	17 (20.5)	10 (12.0)	3 (3.6)	4 (4.8)	0 (0.0)	0 (0.0)	0 (0.0)
Non-cessation Topic (Control Group)								
(*n* = 439)	348 (79.3)	91 (20.7)	47 (10.7)	9 (2.1)	27 (6.2)	4 (0.9)	2 (0.5)	2 (0.5)

**Table 2:** The mean and the SD for the processes applied in the Transtheoretical Model by the combination of comments with reactions.

Processes of Change, Mean (SD)			
Combination of Comment and Reaction			
Non-Combined	Like	Love	Haha
(*n* = 304)	(*n* = 49)	(*n* = 10)	(*n* = 15)
Experiential Processes (EP)			
0.56 (0.63)	0.40 (0.54)	0.50 (0.70)	0.11 (0.33)
Behavioral Processes (BP)			
0.36 (0.55)	0.37 (0.54)	0.80 (0.63)	0.44 (0.53)
All Processes of Change (EP + BP)			
0.61 ^a^ (0.59)	0.63 ^a^ (0.60)	1.10 ^a^ (0.74)	0.33 ^a^ (0.49)
The Proportion of Experiential Processes [EP/(EP + BP)]			
0.61 (0.46)	0.52 (0.48)	0.31 (0.37)	0.20 (0.45)
The Proportion of Behavioral Processes [BP/(EP + BP)]			
0.39 (0.46)	0.48 (0.48)	0.69 (0.37)	0.80 (0.45)

^a^ Significant difference, *p* < 0.05 (2-tailed).

**Table 3:** The mean and the SD of change talk and sustain talk by combinations with reactions.

Motivational Language, Mean (SD)			
Combination of Comment and Reaction			
Non-Combined	Like	Love	Haha
(*n* = 304)	(*n* = 49)	(*n* = 10)	(*n* = 15)
Change Talk (CT)			
1.24 ^b^ (0.99)	1.24 ^b^ (0.78)	2.60 ^b^ (0.97)	0.67 ^b^ (0.72)
Sustain Talk (ST)			
0.54 (0.62)	0.45 (0.77)	0.50 (0.85)	0.80 (0.56)
All Motivational Utterances (CT + ST)			
1.78 ^a^ (1.03)	1.69 ^a^ (1.08)	3.10 ^a^ (1.37)	1.47 ^a^ (0.74)
The Proportion of Change Talk [CT/(CT + ST)]			
0.64 ^a^ (0.42)	0.76 ^a^ (0.37)	0.89 ^a^ (0.19)	0.40 ^a^ (0.44)
The Proportion of Sustain Talk [ST/(CT + ST)]			
0.36 ^a^ (0.42)	0.24 ^a^ (0.37)	0.11 ^a^ (0.19)	0.60 ^a^ (0.44)

^a^ Significant difference, *p* < 0.05 (2-tailed). ^b^ Highly significant difference, *p* < 0.001 (2-tailed).

## Data Availability

Original data in Supplementary Materials File S3. The data sets used and analyzed during the current research are available from the corresponding author on reasonable request.

## Supplementary Materials

The following supporting information can be downloaded at https://www.mdpi.com/article/10.3390/ijerph19169983/s1. File S1: Examples of the investigated stimuli (doc). File S2: Categories of comment analysis (doc). File S3: Original data (xls).
