# Supplementary material for: Smokers’ Engagement Behavior on Facebook: Verbalizing and Visual Expressing the Smoking Cessation Process
Source: Int J Environ Res Public Health. 2022 Aug 12;19(16):9983. doi: 10.3390/ijerph19169983 (PMC9408543; doi:10.3390/ijerph19169983)
Supplement: Supplementary file 1 [file ijerph-19-09983-s001.zip › File S1.pdf]

File S1: Examples of the investigated stimuli

Social media contents which were all image-based, adopted the motivational interviewing principle, and supported smoking cessation.

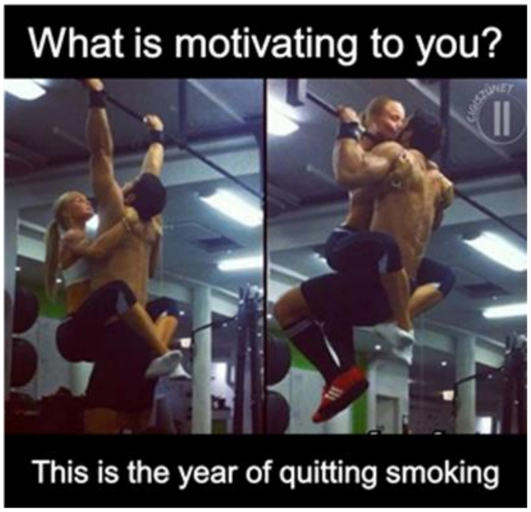

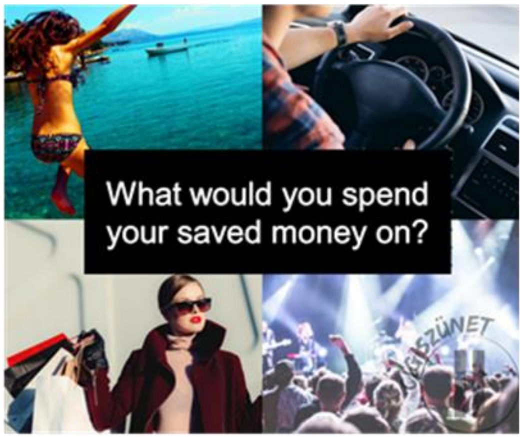

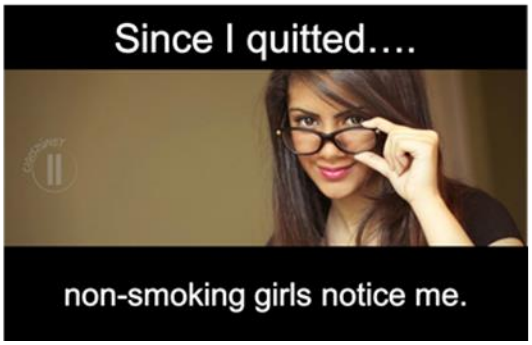

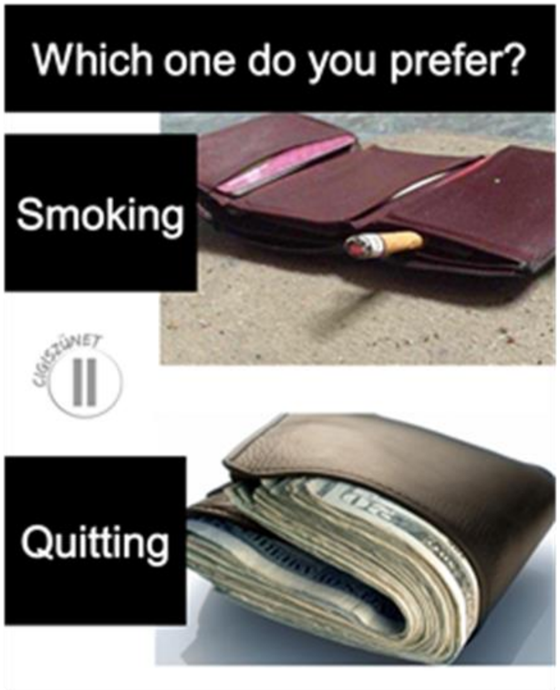

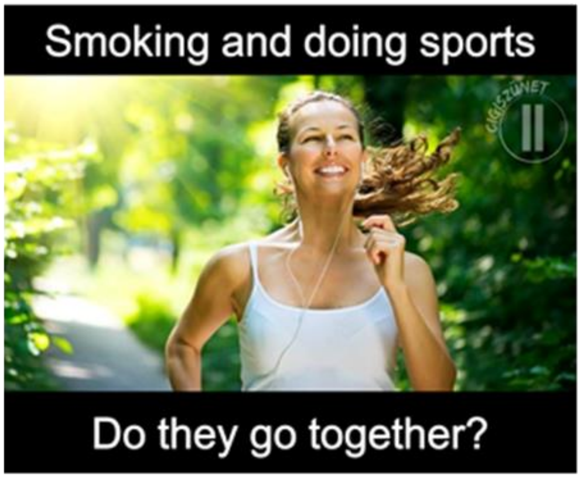

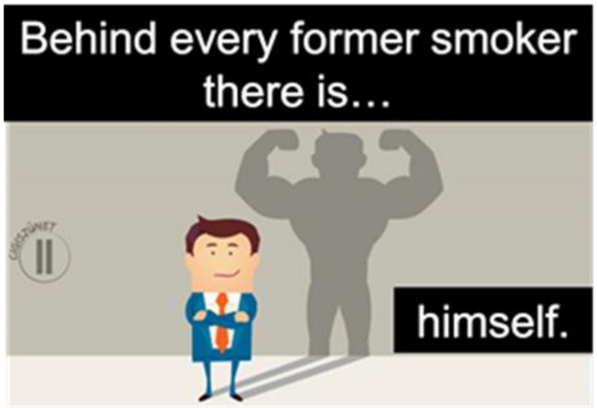
Behind every former smoker there is…
himself.

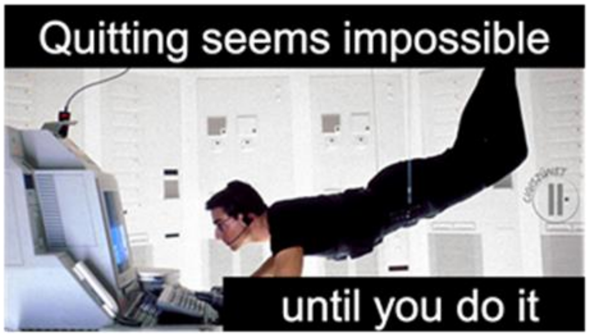
Quitting seems impossible
until you do it

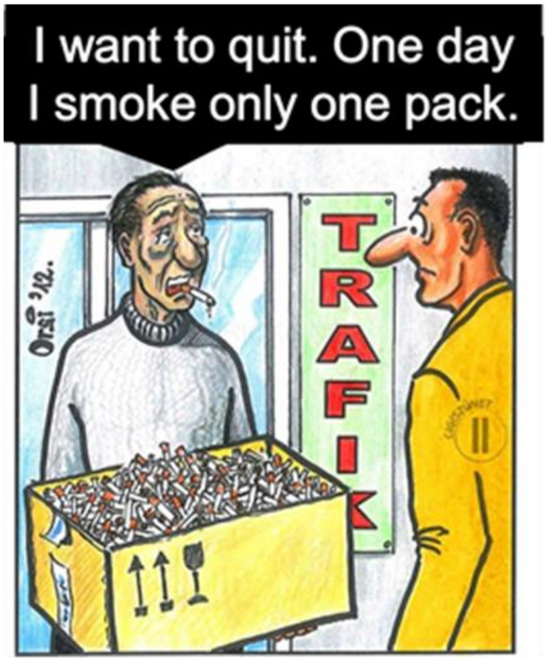
I want to quit. One day I smoke only one pack.
TRAFIK

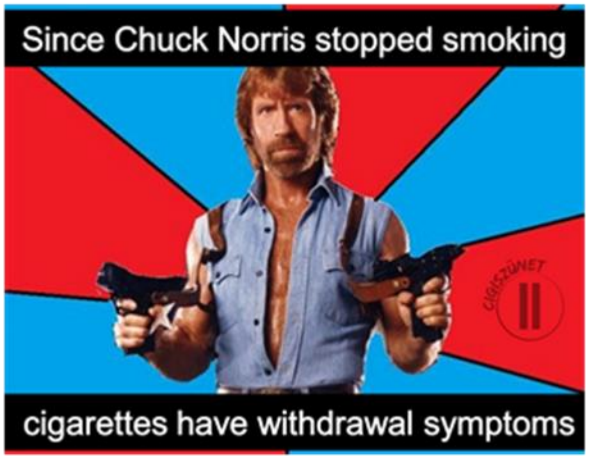
Since Chuck Norris stopped smoking
cigarettes have withdrawal symptoms

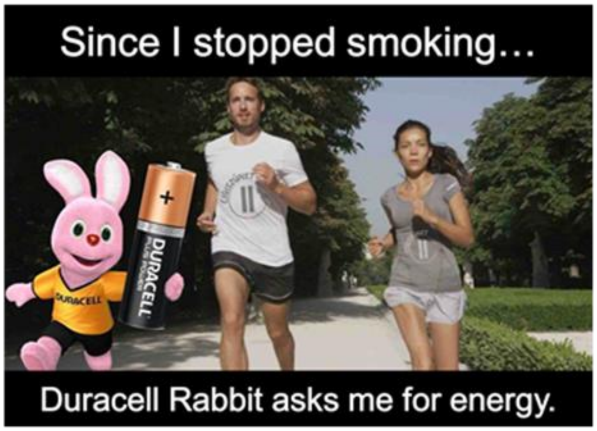
Since I stopped smoking…
DURACELL
Duracell Rabbit asks me for energy.

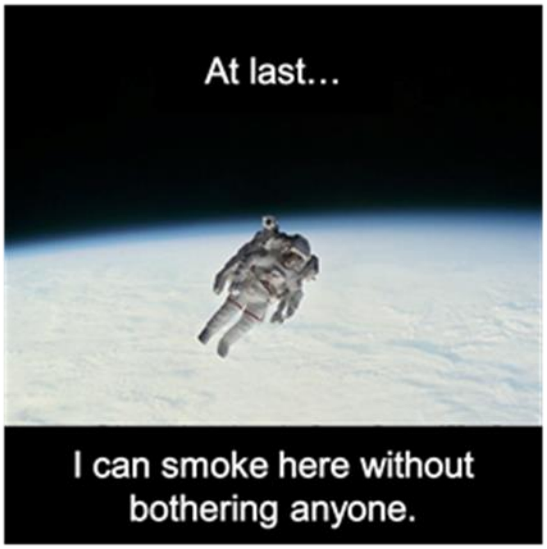
At last…
I can smoke here without bothering anyone.
