# Supplementary material for: Smokers’ Engagement Behavior on Facebook: Verbalizing and Visual Expressing the Smoking Cessation Process
Source: Int J Environ Res Public Health. 2022 Aug 12;19(16):9983. doi: 10.3390/ijerph19169983 (PMC9408543; doi:10.3390/ijerph19169983)
Supplement: Supplementary file 1 [file ijerph-19-09983-s001.zip › File S2.pdf]

File S2: Categories of comment analysis

Processes of change

These phrases are specific actions (processes) which are taken by smokers to stop tobacco use.

Experiential Processes

Smokers' phrases that express cognitive, affective, and evaluative processes are used primarily in the early stage of tobacco use cessation.

Consciousness Raising

Smokers' phrases that increase awareness via any information about tobacco use cessation.

- "I look for information related to my smoking behavior."
- "I think about information from articles and books on how to quit smoking."
- "I read about people who have successfully stopped smoking."
- "I recall information people have personally given me about the benefits of quitting."

Dramatic Relief

Smokers' phrases that express emotional arousal about tobacco cessation.

- "Dramatic portrayals about smoking affect me emotionally."
- "I react emotionally to warnings about the consequences of smoking."
- "Remembering reports about illnesses caused by smoking upsets me."
- "Warnings about hazards of smoking move me emotionally."

Social Liberation

Smokers' phrases that express the recognition that society supports the cessation of tobacco use.

- "I encounter social situations that are designed to help reduce smoking."
- "I find that society is changing in ways that make it easier for me to quit smoking."
- "I notice that people with the same problem I have are asserting their rights."
- "I read about people who have successfully stopped smoking."

Environmental Reevaluation

Smokers' phrases that express the recognition that tobacco use has a negative effect on others, and the cessation of tobacco use has a positive effect on others.

- “I consider the view that smoking can be harmful to the environment.”
- “I feel more empathy for non-smokers who are disturbed by cigarette smoke.”
- “I am angry when I see a smoker who has no regard for secondhand smoke.”
- “I think I can serve as a negative role model as a smoker for my young sister.”

Self-reevaluation

Smokers’ phrases that express the development of a new self-image without tobacco use.

- “I consider that my family and friends would be better off without my smoking behavior.”
- “My tendency to give in to smoking makes me feel disappointed in myself.”
- “I reassess the fact that being content with myself includes quitting.”
- “I get upset when I think about giving in to smoking.”

Behavioral Processes

Smokers’ phrases that express action-oriented processes used primarily in a later stage of tobacco use cessation.

Self-liberation

Smokers’ phrases that express the belief that smokers have the ability to stop tobacco use and are committed to acting on that belief.

- “I tell myself that if I try hard enough, I can stop smoking.”
- “I make commitments against giving in to smoking.”
- “I use willpower to keep from engaging in my smoking behavior.”
- “I tell myself I can choose to quit or not.”

Reinforcement management

Smokers’ phrases that express the rewards for cessation behavior and reduce the rewards for smoking behavior.

- “I do something nice for myself in return for not giving in to smoking.”
- “I counter the temptation to punish myself with covert reinforcements.”
- “I reward myself for small quitting steps.”
- “Other people in my daily life try to make me feel good about smoking cessation.”

Helping Relationship

Smokers’ phrases that express to find people (e.g., family members, friends) who support tobacco use cessation.

- “I have someone who listens when I need to talk about smoking cessation.”
- “I can be open with at least one person about experiences related to quitting.”
- “I have someone on whom I can count when I am craving for cigarettes.”

- ➢ “I have someone who understands my smoking behavior.”

Stimulus Control

Smokers’ phrases that express the use of reminders and signs that encourage cessation behavior and avoid an environment that doesn't.

- ➢ “I remove things from my home that remind me of my smoking behavior.”
- ➢ “I leave places where other people are encouraging smoking.”
- ➢ “I put things around my home or workplace that remind me not to engage in my smoking behavior.”
- ➢ “I relate less often to people who contribute to smoking.”

Counter Conditioning

Smokers’ phrases that express the learning of cessation behaviors that can replace for smoking behaviors.

- ➢ “I engage in some physical activity when I am tempted to engage in my smoking behavior.”
- ➢ “When I feel the craving for cigarettes, I try to relax.”
- ➢ “I find that other activities are a good substitute for smoking.”
- ➢ “When I feel my smoking behavior coming on, I think about or do something else.”

Motivational language, motivational utterances

These phrases used by smokers who express motivation/demotivation for tobacco use cessation or action/inaction towards tobacco use cessation (change talk / sustain talk).

Change talk

Smoker’s phrases that favor movement toward tobacco use cessation.

Preparatory change talk (“DARN”)

Any smoker’s comment that expresses <u>motivation</u> for tobacco use cessation or using tobacco: Desire (e.g., want, wish, like), Ability (e.g., can, could, able), Reason (e.g., specific causes of action), and Need (e.g., need, have to, got to, must).

- ➢ Desire
  - “I want to quit smoking.”
  - “I wish I could quit smoking.”
  - “I like to stop smoking.”
- ➢ Ability
  - “I can quit smoking.”
  - “I could quit smoking.”
  - “I am able to stop smoking.”

- Reasons
  - "I have many reasons to quit."
  - "My health is my motivation to quit smoking."
- Need
  - "I need to quit smoking."
  - "I have to stop smoking."
  - "I must stop smoking."

Mobilizing change talk ("CAT")

Any smoker's comment that expresses <u>action</u> for tobacco use cessation or using tobacco: Commitment (e.g., will, do, going to), Activation (e.g., ready, willing, considering), and Taking steps (e.g., an action already taken).

- Commitment
  - "I will quit smoking."
  - "I quit smoking."
  - "I am going to stop smoking."
- Activation
  - "I am ready to quit smoking."
  - "I am willing to stop smoking."
  - "I am considering quitting smoking."
- Taking steps
  - "I bought a nicotine patch."
  - "I started to reduce smoking."

Sustain talk

Smoker's phrases that favor remaining a tobacco user rather than moving toward tobacco use cessation.

- "I don't want to quit smoking."
- "I wish I could smoke again."
- "I can't quit smoking."
- "I couldn't quit smoking during pregnancy."
- "I won't be able to stop smoking."
- "I've got no reason to quit."
- "I have many reasons to smoke cigarettes again."
- "I need to smoke."
- "I don't have to quit smoking."
- "I mustn't quit."
- "I will smoke again."
- "I am not ready to quit smoking."
- "I'm considering smoking again."
- "I bought a pack of cigarette."
- "I smoked my friend's cigarette."
